# The value of mean platelet volume in the determination of community acquired pneumonia in children

**DOI:** 10.1186/1824-7288-39-16

**Published:** 2013-03-08

**Authors:** Eda Karadag-Oncel, Yasemin Ozsurekci, Ates Kara, Sevilay Karahan, Ali Bulent Cengiz, Mehmet Ceyhan

**Affiliations:** 1 Hacettepe University Faculty of Medicine, Pediatric Infectious Disease Unit, Ankara, Turkey; 2 Department of Bioistatistics, Hacettepe University Faculty of Medicine, Ankara, Turkey

**Keywords:** Community-acquired pneumonia, Child, Mean platelet volume, Severity

## Abstract

**Background:**

Mean platelet volume (MPV) is a reflection of platelet size, which has been shown to correlate with platelet function and activation. The aim of this study was to evaluate whether MPV could be used for the diagnostic tool of community-acquired pneumonia (CAP) and for making the decision for hospitalization.

**Methods:**

The computerized records of children aged 1 to 18 years who were diagnosed with CAP based on WHO criteria were evaluated. A standard protocol was followed, and patients with severe CAP were hospitalized. CAP patients were divided into two groups based on disease severity. The control group consisted of age and gender matched healthy children during the study period. Values for hemoglobin, white blood cell count (WBC), platelet count, MPV and C-reactive protein (CRP) obtained on first presentation were recorded for each patient.

**Results:**

A total of 196 patients were diagnosed with CAP during the study period, 108 (55.1%) of which had severe disease, which required hospitalization (Group 1a), while the remaining 88 (44.9%) were followed-up as outpatients (Group 1b). The control group consisted of 100 healthy children (Group 2). Patients with CAP had lower MPV values than their healthy counterparts (7.1±0.68 vs. 8.31±1.2 fL; p<0.001). MPV value was significantly higher in hospitalized CAP patients compared to outpatients (7.32±0.71 vs. 6.83±0.5 fL; p=0.012). ROC curve analysis suggested that MPV level cut-off point for making a diagnosis of CAP was 8.1 fL, with a sensitivity, specificity, positive predictive value (PPV), and negative predictive value (NPV) of 91%, 51%, 80.8% and 70.5%, respectively.

**Conclusions:**

Our findings suggest that MPV may be a useful predictor for diagnosed CAP but low specificity and NPV rates may lead to the false-negative diagnosis.

## Introduction

Community-acquired pneumonia (CAP) is a serious and frequent cause of hospital admission in early childhood [1] In developing countries, the incidence of childhood hospitalizations due to CAP is estimated to be 8.7% of all cases of CAP [2], whereas the hospitalization rates for children under the age of 5 years in the UK and USA are 2.9/1000 [3] and 6.6/1000 [4], respectively. The clinical spectrum of CAP in children ranges from mild disease, which can be managed on an outpatient basis, to a severe life-threatening condition requiring prolonged intensive care [1].

Many biochemical markers have been investigated in association with clinical outcome in patients with CAP, including several cytokines such as tumour necrosis factor-α and interleukin 6 (IL-6), C-reactive protein (CRP), procalcitonin, and D-dimer. The role of cytokines as important mediators of lung defence against infections and inflammation is well documented. Cytokines may have either pro- or anti-inflammatory effects depending on a multiple of interacting microbiologic, environmental, and genetic factors that are believed to influence host response to respiratory infections [5-8].

There is a growing body of clinical evidence suggesting that platelets play an important role in the inflammatory response. Multiple inflammatory factors such as chemokines, cytokines and coagulation factors are secreted by platelets, which increase in size when they are activated. Mean platelet volume (MPV) is a reflection of platelet size, which has been shown to correlate with platelet function and activation. A higher MPV value is indicative of increased platelet activity and thus more intense inflammation [9]. Changes in MPV have been studied in several chronic inflammatory diseases [10-14], however, to the best of our knowledge, such changes have not been previously studied in association with CAP. The aim of our study was to investigate whether MPV values are affected by the inflammatory response in childhood CAP.

## Materials and methods

### Study design

This study was approved by the local ethics committee in accordance with the Helsinki Declaration. Informed consent was obtained from the parent or legal guardian of each participant. This retrospective study was conducted in Ihsan Dogramaci Children’s Hospital of the Faculty of Medicine at Hacettepe University, which serves as a referral tertiary care center for all Anatolia. The medical records of children between 1–18 years of age who were diagnosed with CAP at the Department of Pediatric Infectious Diseases between January 2010 and June 2012 were systematically reviewed. Excluded criteria were presence of underlying chronic disease, history of previous hospitalization, any blood diseases or anemia. In all patients, a diagnosis of pneumonia was made based on the case definition proposed by the World Health Organization (WHO); any child presenting with cough or breathing difficulties was considered eligible if they had tachypnea (>40/min, age 12–59 months; >30/min, age ≥60 months) or in drawing without wheeze, as well as having an abnormal chest radiograph (consolidation or perihilar infiltrates) [15]. Patients with severe CAP, as defined by WHO [16] and British Thoracic Society [17] criteria, were hospitalized. The control group consisted of healthy age and gender-matched children who attended the “Well-child” clinic for routine check-up in the same period.

Information regarding demographics, medical history, clinical characteristics and laboratory results were retrieved from computerized hospital medical records. Complete blood counts were performed for all patients on presentation using a commercially available analyzer (Sysmex XT 2000i, Roche Diagnostics GmnH, Mannheim, Germany). Hemoglobin level, white blood cell count (WBC), platelet count, and MPV values were recorded for each patient. The reference range for MPV was between 7.0 and 11 fL. All CAP patients also had baseline CRP levels available. Chest radiographs of the study population, which were retrieved from the hospital’s database, were re-evaluated by a designated pediatric radiologist for the presence of signs supporting a diagnosis of CAP.

### Statistical analyses

Statistical analyses were performed using the Statistical Package for Social Sciences version 17.0 (SPSS for Windows 17.0, Inc., Chicago, IL, USA). Categorical variables were provided as numbers and percentages, whereas numerical variables were given as mean ± standard deviation. Gender comparisons between groups were made using the Chi-square test. Initial comparisons of laboratory parameters between the three groups were made using either the Welch analysis of variance (ANOVA) test or the Kruskal Wallis test, depending on normality of distribution, followed by two-way comparisons using either the *t*-test for independent samples or the Mann–Whitney test, respectively. The cut-off value for MPV that best distinguishes between healthy controls and CAP patients were determined using ROC analysis, for which sensitivity and specificity values were calculated. A *p*-value of less than 0.05 was considered indicative of statistical significance.

## Results

A total of 196 patients were diagnosed with CAP in the department of Pediatric Infectious Diseases, 108 (55.1%) of which required hospitalization (Group 1a), while the remaining 88 (44.9%) were followed-up on an outpatient basis (Group 1b) with oral antibiotics. The control group (Group 2) consisted of 100 children without an underlying chronic disorder who underwent blood workup during routine check-up. The mean age of all participants was 6.8±4.3 years (Group 1a, 6.9±3.9 years; Group 1b, 6.6±4.8 years, Group 2, 6.9±4.3 years), with an overall gender distribution of 52% males and 48% females. The difference between the three groups with regards to mean age (p=0.93) and gender distribution (p=0.22) was statistically insignificant.

**Figure 1:**
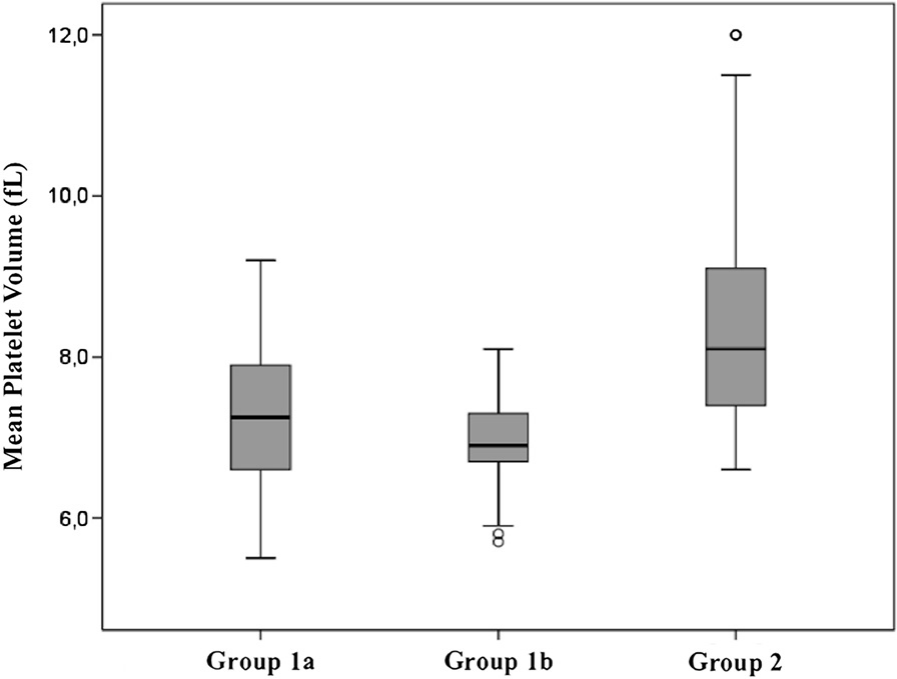
**Comparison of MPV values in Group 1a** (**n**=**108**), **Group 1b** (**n**=**88**) **and Group 2** (**n**=**100**)**.**

Patients with CAP had a significantly lower mean MPV value compared to healthy controls (7.1±0.68 vs. 8.31±1.2 fL; p<0.001). ROC curve analysis suggested that MPV level cut-off point for making a diagnosis of CAP was 8.1 fL, with a sensitivity, specificity, positive predictive value (PPV), and negative predictive value (NPV) of 91%, 51%, 80.8% and 70.5%, respectively (Area under curve: 0.796). MPV value was significantly higher in hospitalized CAP patients compared to outpatients (7.32±0.71 vs. 6.83±0.5 fL; p=0.012). MPV values of groups are depicted in Figure 1.

WBC levels were significantly higher in CAP patients who were hospitalized (p<0.001) or followed-up as outpatients (p<0.001) than healthy controls. CRP levels were significantly higher in CAP patients who were hospitalized compared to those who were followed-up as outpatients (p<0.001). A summary of laboratory results of the study population is provided in Table 1.

**Table 1:** Comparison of laboratory parameters of study population

**Parameter**	**Group 1a (hospitalized) (n**=**108)**	**Group 1b (outpatient) (n**=**88)**	**Group 2 (control) (n**=**100)**	***p***-**value**
**Hb (/gL)**	121±16	124±14	128±11	NS^a,c^ 0.05^b^
**WBC (10**^**9**^**/L)**	13100 [2500–50500]	9800 [2500–24000]	7900 [4100–12700]	0.019^a^ <0.001^b,c^
**Platelet count (10**^**9**^**/L)**	34610±151365	335110±128930	321805±79030	NS^a,b,c^
**CRP (mg/dL)**	3.98 [0.2-42]	0.8 [0.1-6.2]	-	<0.001^a^
**MPV (fL)**	7.32±0.71	6.83±0.5	8.31±1.2	0.012^a^ <0.001^b,c^

^a^Group 1a versus Group 1b, ^b^Group 1a versus Group 2, ^c^Group 1b versus Group 2.

Variables showed Mean±SD or median [min-max]; Hb, hemoglobin; WBC, white blood cell count; CRP, C-reactive protein; MPV, mean platelet volume; NS, not significant.

## Discussion

Platelet volume, which is determined at the level of progenitor cells (i.e. the megakaryocyte), is correlated with platelet function and activation [18]. The role of MPV as an indicator of platelet function has been investigated in association with several inflammatory disorders such as cystic fibrosis [10], ulcerative colitis [11], rheumatoid arthritis [12], familial Mediterranean fever (FMF) [13], neonatal respiratory distress syndrome [14] and infections such as upper urinary tract infections [19] and sepsis [20,21]. In previous studies, it has been suggested that cytokines such as IL-3 and IL-6 may influence megakaryocyte ploidy leading to the production of larger and more reactive platelets [22,23]. IL-6, which plays a central role in the inflammatory process of sepsis and CAP, is also believed to affect MPV. The relationship between MPV and sepsis has also been evaluated in a number of studies [20,24]. Becchi et al. [20] reported on higher mean MPV values in patients who survived sepsis compared to those who did not survive. In a study on children with CAP, out of 15 different cytokines investigated, a significant correlation with disease severity could only be observed with IL-6 levels [25]. These studies may help explain the lower MPV values we observed in CAP patients compared to healthy controls. Logistic regression analysis revealed an MPV value of less than 8.1 on presentation to have a sensitivity of 91%, a specificity of 51%, a PPV of 80.8% and a NPV of 70.5% for CAP diagnosis. The low specificity and NPV rates may lead to the false-negative diagnosis.

To the best of our knowledge, this is the first study in which the role of MPV as a diagnostic and prognostic factor for CAP has been evaluated. In our study patients with more severe disease that required hospitalization were found to have significantly higher MPV values compared to patients who were followed-up on an outpatient basis (p=0.012). The lower MPV values observed in patients followed-up on an outpatient basis are an interesting finding that is not interpretable based on current knowledge. It may be hypothesized that the bone marrow response to the infection is inadequate in CAP cases followed as outpatients, and that an increase in platelet size only occurs as a result of damage or consumption of peripheral circulating platelets due hyper stimulation. Considering that patients who require hospitalization tend to be brought to the hospital at a later stage of the disease, it may be postulated that MPV decreases during the earlier stages of CAP, which is followed by a significant increase in MPV as a result of bone marrow activation.

Pneumonia can generally be accepted as an infectious inflammatory process of the lungs. There are some studies that varied MPV levels in certain inflammatory conditions. Studies conducted in children, in regard of control groups MPV decreased significantly in acute exacerbation period of cystic fibrosis [10], Kawasaki disease with coronary artery lesion [26], acute attack period of FMF on colchicine treatment [13]. In regard of control groups MPV increased significantly during acute attack and attack-free period of pediatric FMF [27], in adult FMF [28], adult active tuberculosis which decreased significantly with anti-tuberculous therapy [29]. Tuncel et al. [30] in a recent study demonstrated that MPV values in asthmatic children both during an asthmatic attack and during the asymptomatic period had no statistically significant difference compared to the healthy control group. And they found that no statistically significant difference between mean MPV values in asthma exacerbation and asymptomatic period. An increased MPV levels (unrelated with platelet count and the type of cultured microorganisms) was found in 13 of 25 patients with culture proven septicemia but none of control group (localized bacterial infection and negative blood cultures). After one week of treatment MPV was normalized [24]. Robbins et al. [31] made a comment about the MPV and infection relationship. They concluded that at least two patterns of platelet size changes could be possible in response to infection: an early rise in MPV in severe infection such as septicemia (may be associated with thrombocytopenia) and a later decrease in MPV with sustained or chronic or persisted bacterial infection (may be associated with thrombocytosis). However, we believe that future prospective studies may help elucidate whether MPV could be used in the follow-up of CAP patients, particularly for the evaluation of response of treatment.

Some studies reported that CRP was associated with disease severity and mortality in hospitalized patients with CAP [5,32]. This is not our main purpose but in our study by comparing CRP and WBC levels of hospitalized CAP patients and those followed-up on an outpatient basis, we managed to demonstrate a statistically significant correlation between CRP and WBC levels and disease severity.

Our study has some limitations because of its retrospective nature. Therefore we could not calculate the time interval between the onset of symptoms and blood sampling. If we achieve this data, we could explain more clearly the difference between MPV values in hospitalized patients and outpatients.

In conclusion, MPV is a simple parameter that is provided by most commercially available automated hema-tology analyzers, with no associated extra cost or effort. Our findings suggest that MPV may be a useful predictor for diagnosed CAP but not in disease severity. However clinicians should be aware of the low specificity (51%) and NPV (70.5%) rates that may lead to the false-negative diagnosis. Further prospective studies on a larger patient population are required to establish the exact role of MPV in patients with CAP, either as an indicator of disease severity or for the evaluation of response to treatment.

## Consent

Written informed consent was obtained from the patient and/or parents for publication of this report and any accompanying images.

## Abbreviations

MPV: Mean platelet volume; CAP: Community-acquired pneumonia; WHO: World Health Organization; WBC: White blood cell count; CRP: C-reactive protein; IL-6: Interleukin 6; IL-3: Interleukin 3; FMF: Familial Mediterranean fever.

## Competing interests

The authors declare that they have no competing interests.

## Authors’ contributions

EKO, YO and AK designed, conducted and analyzed the study; SK analyzed the statistical data, all authors were involved in the primary care of the patient. All authors have received and approved this manuscript.
